# Mood, Delusions and Poetry: Emotional ‘Wording of the World’ in Psychosis, Philosophy and the Everyday

**DOI:** 10.1007/s11406-017-9854-8

**Published:** 2017-05-27

**Authors:** Owen Earnshaw

**Affiliations:** Department of Philosophy, Durham University, 50 Old Elvet, Durham, DH1 3HN UK

**Keywords:** Delusions, Poetry, Attunement, Mood, Emotions, Heidegger

## Abstract

Starting from a comparison of the similarities between a poem by Sylvia Plath called *Tulips* and the words of someone in the thrall of a delusion I develop a phenomenology of how mood is basic to our articulation of the world. To develop this argument I draw on Heidegger’s (1962) concept of attunement [befindlichkeit] and his contention that basic emotions open up aspects of the world for closer inspection and articulation. My thesis in this paper is that there is an underlying structural similarity between the forms of words used in poems and those found in medically diagnosed delusions and this similarity is based on the role of mood in both arenas. The difference, I argue, is that although both forms of articulation are negotiated ‘as if’ the subject matter was literal, the person writing the poem is self-aware that their uses of language are figurative and metaphorical. This is because the emotional lens they use to describe a situation poetically can always be removed by a return to a ground-mood of acceptance, that prevents them from becoming lost in the poetical mood. The person experiencing psychosis, on the other hand, is unable to extricate herself from the mood that underlies their delusional utterances as they have lost access to the ground-mood that the poet takes for granted. I illustrate the point using Hume’s famous statement about the mood he philosophises in and look at ways sufferers from delusions could regain a sense of the non-literal projections of their words.

I will start with a quote. It is an excerpt from a poem by Sylvia Plath (1965) in her collection *Ariel.* The poem is called *Tulips*:

The tulips are too red in the first place, they hurt me. Even through the gift paper I could hear them breathe Lightly, through their white swaddlings, like an awful baby.Their redness talks to my wound, it corresponds.They are subtle: they seem to float, though they weigh me down,Upsetting me with their sudden tongues and colour,A dozen red lead sinkers round my neck.

Nobody watched me before, now I am watched.The tulips turn to me, and the window behind meWhere once a day the light slowly widens and slowly thins,And I see myself, flat, ridiculous, a cut-paper shadowBetween the eye of the sun and the eyes of the tulips,And I have no face, I have wanted to efface myself.The vivid tulips eat my oxygen (Plath 1965).

The poem these lines are taken from is a very sad, beautiful evocation of a time Plath spent in hospital. However, if these lines are read under the aspect of a literal description of that time, they suddenly appear like the words of someone in the thrall of a paranoid delusion (e.g. that the tulips hurt her, the personification of the tulips as breathing, the references to the tulips watching her and the thought that she has no face). This could be compared with words from a woman who has experienced delusions called Esso Leete^1^:

It was evening and I was walking along the beach near my college in Florida. Suddenly my perceptions shifted. The intensifying wind became an omen of something terrible. I could feel it becoming stronger and stronger; I was sure it was going to capture me and sweep me away with it. Nearby trees bent threateningly toward me and tumbleweed chased me. I became very frightened and began to run. However, though I knew I was running, I was making no progress. I seemed suspended in space and time (Torrey 2001).

Here we can see a similar perception of the environment as animated. The obvious difference is that Plath is writing from an ‘as if’ stance (by which I mean she is writing ‘as if’ these the plants were watching her but is able to step back into commonsense after writing), whereas the delusion of Leete seems to be a literal apprehension of the world, in that it guides her behaviour and her emotional reaction to the situation.

In this paper I will outline a theoretical understanding of the similarities between poetry and delusions and explain why poets are self-aware of what they are doing with words whereas sufferers from delusions are not. I will do this in terms of the background structure provided by moods and argue that, crucially, people with delusions do not have access to the ground-mood of acceptance that allows us to fall into other moods without becoming lost in them.^2^ I will then go on to treat the subject of how moods can be seen to operate when philosophising to illustrate my position. I will look at Wittgenstein’s conception of philosophy as language going on holiday to back up my claims. Next I analyse a competing theory of delusions by Currie and Jureidini (2001) that conceives delusions as ‘imaginings’ that are mistaken for beliefs and show how my position can encompass their claims and is phenomenologically richer while doing the same explanatory work. Finally I will briefly survey what this conception of the role of moods in delusions implies about the treatment of those suffering from delusions and examine ways we can use this understanding to lead words back from a ‘metaphysical’ use to an everyday use.

## Mood and the ‘as if’ Stance

One possible way to understand poetical discourse is given by Heidegger in *Being and Time* (1962)*.* He claims:

In ‘poetical’ discourse, the communication of the existential possibilities of one’s state-of-mind [Befindlichkeit] can become an aim in itself, and this amounts to a disclosing of existence (Heidegger 1962).

‘State-of-mind’ is an unfortunate translation of Befindlichkeit as it suggests that he is referring to a private mental state, which is what he wanted to avoid. ‘Attunement’ better emphasises that Heidegger is putting forward the idea that our mooded apprehension of the world underpins and gives sense to our cognition of the world. To summarise his position, he argues that we are rooted and orientated in the world by the way it ‘matters’ to us (e.g. through moods (Heidegger 1962)). Heidegger argues that the way the world matters to us is a basic framework within which cognition operates (Heidegger 1962). One way of understanding this is by highlighting that moods provide the background on which anything can show up as significant and worthy of attention, which is the point at which cognition comes into play.

A more contentious claim that Heidegger can be understood to be making is that moods actually open up for us a way the world really is, rather than being merely a subjective colouring to a scientifically-described, physical world.^3^ This claim was also made by Wittgenstein when he wrote that, ‘the world of the happy man is a different one from that of the unhappy man’ (Wittgenstein 1974). A possible way of cashing out this counterintuitive approach to affect is to say that the fact of the world as the place we inhabit (as creatures with certain drives and needs) is the ground on which all our other ways of making sense of it (e.g. through science) rest. In other words, the interpersonally constituted world, with its many and various layers of culture and meaning, is not a secondary or deficient mode of grasping the world to the way the world is understood through abstract contemplation in the form of science. If this is accepted then it can be argued that moods open this world up for our inhabitation both by attuning us with others and by constituting which objects in the environment appear meaningful and thereby salient to us. In this sense, our attunement to the world means that moods can be said to constitute our objective environment, rather than just being ‘inner’ states.^4^

This claim is backed up by contemporary work in the philosophy of emotions. In order to interact with the world, according to theorists such as Gigerenzer and Hookway, we employ heuristics or ‘gut-feelings’ (Gigerenzer 2007) that, on the whole, allow us to operate in the world efficiently without going through immense amounts of computation. (Hookway 2002). That is, we use our moods as a basis for cognition of the world. As the term ‘gut-feelings’ indicates the claim is that our inquiries into the natural world are regulated through our emotional and mooded engagement with that same world. This position is taken further in Gibson’s (1986) notion of *affordances*, where an emotion:

[R]efers to a relational property, the mode of presentation consists in an action orientation that makes the property appear in a certain way, in the case of fear something dangerous appears as something to be avoided (Hufendiek 2016).

So the person perceives a feature or features of the environment in terms of their own abilities or action-readiness. This implies that the objective world is constituted and perceived as being an environment in which the properties of the world are related to the behavior and the behavior-readiness of the person. This highlights that the background structure of moods and emotions determines how we can perceive the world and different moods and basic emotions will open up different realities for us. For example science may be understood as having to be carried out in a mood of cool detachment in order for the results to be generally applicable whereas working for social justice requires a mood of concerned engagement that relies on the human capacity for empathy and kindness. This may be an oversimplification (and the scope of the essay does not allow me to go into more detailed argumentation here) but the fact that moods and emotions underlie our cognition is strongly suggested by the point made above that our environment is constituted by our abilities and our action orientation and we rely on emotions to make certain properties salient and meaningful for us. A change in basic emotion therefore would dictate a change in how we perceive the environment e.g. as hostile, welcoming, boring etc. and also what story we tell about the that environment. If we are driven by a threat to our person the environment itself becomes threatening and we look for ways to avoid the impending attack. An overactive threat system makes everything seem potentially harmful and the narratives we weave about our situation would thereby become based on this new way of looking at the world. This way of looking at the world is not a mere ‘subjective colouring’, but rather is the world showing itself in relation to our needs and concerns as an organism. This conception of the role of emotions should be seen as a sketch fleshing out Heidegger’s claim about mood and I hope this will have given at least some reasons to accept this point before developing the claim further.

The point of this digression into Heideggerian ontology is to try to identify the aspect in which poetry can be seen as similar to delusions. Taking Heidegger’s quote, alongside the elaboration of the idea of attunement, the link between poetry and delusions can be seen as the commonality of focus on the expression of an underlying mood that permeates the person’s inhabitation of the world. A feature of art in trying to accurately express a mood is that (as is found by poets) only a certain ordering of words will do to express the particular mood. For both the poet and the delusional person only certain words will do, in the same way that poems are un-paraphrasable. By this I am simply pointing out the fact that an exegesis of a poem cannot capture the aesthetic effect of the poem itself, but rather must explain what those particular words, in that particular, order do to the reader through the particular language used. We can make this point (again using a Heideggerian concept) by saying that these words are ‘ready-to-hand’ for the delusional person. The idea here is that everyday orderings of words just do not capture the mood the person is trying to communicate (because the mood is completely ‘out of the ordinary’) and so new ways of talking about objects and situations are required. What is being attempted is an expression of the mood through which the person is inhabiting the world. In both cases we could try and identify the mood the person is trying to capture for example we might say that in Plath’s case, she is elaborating a despairing tranquillity and in Leete’s case, an ominous foreboding. This would, however, precisely miss the point that only the particularity of the words they actually use really expresses the mood they inhabit.

The ‘as if’ stance of Plath can be seen as a sense in which she is aware of what she is attempting through words, whereas for Leete the mood is so basic and all-encompassing that there is no space between the sense she makes of the mood and her general orientation in the world. For Leete there is no recourse to the basic mood of acceptance, where the person has a strong sense of confidence in the commonsense everyday understanding of the world from an objective viewpoint, from which she could contemplate the mood that underlies the delusion. I would contend that it is the lack of the basic atmosphere of acceptance that would allow a contemplative stance (or what I have been calling the ‘as if’ stance in writing poetry) which is the element that separates the language-game of poetry from that of the delusional person’s expression of a (mood inflected) world from which they are unable to escape.^5^ Acceptance can be taken as an underlying ground mood that allows us to fall into other moods without becoming completely lost in them.^6^ In other words, in the case of a delusion there is no fall back position from which to extricate themselves from the all-encompassing mood of the delusion. In the case of poetry, the mood that is expressed can always be dispelled through a return to the everyday atmosphere of acceptance, which is still available to the person writing the poetry.

## Language ‘on Holiday’

An example of mood playing a part in our ‘wording of the world’ (Cavell 1989) can also be found in philosophy, most famously in the writing of Hume. He describes a particular reverie that he finds himself in when philosophising:

I am confounded with all these questions, and begin to fancy myself in the most deplorable condition imaginable, inviron’d with the deepest darkness, and utterly deprived of the use of every member and faculty.

Most fortunately it happens, that since reason is incapable of dispelling these clouds, nature herself suffices to that purpose, and cures me of this philosophical melancholy and delirium, either by relaxing this bent of mind, or by some avocation, and lively impression of the senses, which obliterate all these chimeras. I dine, I play a game of back-gammon, I converse, and am merry with my friends; and when after three or four hour’s amusement, I wou’d return to these speculations, they appear so cold and strain’d and ridiculous, that I cannot find in my heart to enter into them farther (Hume 1978).

In this example Hume identifies a certain mood that goes with his philosophising, a melancholy or delirium that he dispels by returning to the social world. This illustrates the idea I put forward above that a ground-mood of acceptance allows a person to return from a mood in which the world seems altered from the everyday commonsense world. The mood in this case could be labelled ‘Sceptical’ and it might be suggested that the product of trying to find words for such a mood can be found arrayed through much of the literature of modern philosophy starting with Descartes. I should emphasize that this claim is not saying that philosophy is merely bad poetry, rather, as I suggested above, it is helpful to understand attunement as opening up for us aspects of the world and certain aspects of reality that may only be available in the Sceptical mood. Descartes himself states that if he were to announce seriously that he is willing to doubt whether the hands before him are his own, as dictated by his sceptical method, then he would be taken for a madman (Descartes 1988). This acknowledges that without the ‘as if’ stance the ideas of philosophers would often be taken for delusions. This is something Sass (1994) hints at when looking at the writings of Schreber through Wittgenstein’s analysis of solipsism. Solipsism attested to without the ‘as if’ stance would undoubtedly be considered delusional, but as with scepticism it can seem the unavoidable conclusion of a philosophical meditation. It is a typical experience for a philosopher to sometimes feel that ordinary everyday ways of orientating in the world must be wrong and that thinking has lead them to some new revelation about how the world really is. This, of course, has obvious parallels with the onset of delusions with the exception that such a thought as solipsism can usually be forgotten through some distraction found in everyday activities or socialising, because, as Hume says, the cure is in our natural habits.

To extend the analogy between philosophy and delusions, it might be helpful to look at Wittgenstein’s claim that, ‘philosophical problems arise when language *goes on holiday*’ (Wittgenstein 1963). By this, I think he means that philosophical problems arise when words are no longer being projected in their familiar everyday sense. The idea that language has left the everyday suggests, firstly, that it is not doing any ‘work’ but also that it has left home for a distant land. The first sense might reflect the idea that, as with poetry, philosophy tends to deal with matters detached from everyday practical concerns. The second sense could be taken to mean that there are realms that can only be charted from this self-imposed exile from the everyday. I do not think that the first sense is necessarily a damning criticism of such uses, for instance, one way of understanding ‘holiday’ is that of being in a mood of relaxed enjoyment and of course in contemporary society a holiday is taken to ‘recharge the batteries’, that is, to return to the everyday with a new appreciation of life. From this it could be said that philosophy happens when we take up a view on life from the perspective of a mood that differs from our normal practically concerned one and which enables us to return with a better sense of what is important. Although this may seem an unwarranted elaboration of a passing comment by Wittgenstein, it is arguable that language going on holiday is an apt way of describing delusions. It is perhaps Wittgenstein’s sense of how close philosophising can be to insanity that encouraged him to use the idea of ‘therapy’ as a name for what he was trying to achieve in his own work. The warning that is inherent in Wittgenstein assertions in *Philosophical Investigations* is that if we do not remind ourselves of the primary projections of our words and their ‘home’ in the everyday we risk becoming entangled in the new pictures of reality we create. If, as I have argued, the everyday sense of the world requires an atmosphere of acceptance then this warning may capture the way the person suffering from delusions becomes lost in their words and may suggest a possible route home, that of being reminded of the everyday projections of words.

## Delusions as Imaginings

At this point it is necessary to contrast the position I have been developing with one that can seem markedly similar, namely Currie and Jureidini’s theory (2001) that delusions are ‘imaginings’ that are mistakenly taken for beliefs by the person with the delusions. The similarity of their theory to my account could be articulated as the way people suffering from delusions take what are merely flights of the imagination in poets, namely ‘imaginings’, to be beliefs. In other words, the ‘as if’ stance that contains ‘imaginings’ outside of the realm of actual belief, is not available for the delusional person to adopt and so there is no boundary between imaginings and beliefs. The attraction of their theory is that it explains not only the reason why the delusion is not questioned, but also why certain unusual experiences should give rise to what seem to be beliefs that contradict a lot of other beliefs the person has. The relevant traits of an imagining are that it is ‘much more easily triggered by perception than is belief’ (Currie and Jureidini 2001) and ‘it is surely quite common to imagine all sorts of wild hypotheses in response to an odd experience’ (ibid.). As well as this, with imaginings there is not the same pressure to resolve the tension with other beliefs. In other words, there is a natural ‘suspension of disbelief’ when imagining. Finally, they are not normally revised in the light of evidence as the main point of imaginings is that they deal with the non-actual.

The main problem with their theory comes when they try to account for why someone should mistake an imagining for a belief. They put this down to some, as yet unknown, ‘sub-personal capacity’ (ibid.) that is damaged and thereby rule out the possibility of empathy. Empathy, they argue, is impossible because, in the same way as it would be impossible to empathise with someone who had lost the capacity to recognise people by their faces because of brain damage, it is impossible for us to imagine what it would be like to mistake a belief for an imagining because our sub-personal capacity is intact. The first problem with this is that it rules out a priori the possibility of empathising with the person suffering from delusions, which is a way of denying community with actual people without good reason. The need to acknowledge a person as an equal partner in conversation necessitates an attitude of taking the possibility of finding sense in a delusion as being an empirical matter to be explored through conversation. The second problem, is that this a priori claim is based on a hypothesis that there is some underlying damage to the brain that is as yet undiscovered. The contrast with my account using Heidegger’s concept of attunement is that my position does not require the positing of such damage and thereby keeps open the possibility of empathising with the person with delusions.^7^

If we ignore the claim about the mistaking of imaginings as beliefs as being due to damage to a sub-personal capacity and look at the phenomenological claims then their account seems to be on firmer ground. They claim that, ‘the deluded person fails to monitor the self-generatedness of her imagining that P’ (ibid.) and so the imagining can come to seem like it is generated by their contact with the world in the normal way that beliefs are. This idea that imaginings are taken for beliefs because of their changed phenomenology can be seen to fit with the picture of psychosis that I have been developing. Whereas I talk about an ‘as-if’ stance in poetry that can only be taken if there is an underlying mood of acceptance to return to (which is not possible in the case of delusions), they talk about imaginings having the same phenomenological character as beliefs. My account can encompass their claim by positing that delusions arise because the mood of acceptance is not available from which to discriminate imaginings as imaginings through a commonsense orientation to the world. In other words, imaginings and beliefs take on a similar phenomenological character because the person is engulfed in an all-encompassing mood. I believe that my account provides a richer phenomenological context that can show how their account is possible without needing to posit damage to a sub-personal capacity. To sum up what I have been saying, delusions can be seen as attempts to express a mode of attunement as a picture of the reality the person suffering from delusions inhabits (in the way I have argued poets and philosophers do). However, the person with the delusion is unable to return to the everyday mood of basic acceptance from which to understand the expression in terms of an ‘as-if’ stance.

## Returning Words to the Everyday

The depiction of delusions as expressions of a mooded apprehension of the world (explored above) suggests avenues for both empathising with the delusional person and also possible ways for overcoming delusions. Central to this understanding of delusions is the way that a return to the everyday requires the return to an atmosphere of acceptance. In the arguments above I focussed on the way that mood influences the ‘ready-to-hand’ nature of particular words that are involved in creating new pictures of reality. However, the relation between mood and words is not a one-way street and of course particular orderings of words can in their turn induce moods in the audience which is part of the attraction of reading poetry. As Heidegger states:

Publicness, as the kind of Being which belongs to the ‘they’…not only has in general its own way of having a mood, but needs moods and ‘makes’ them for itself. It is into such a mood and out of such a mood an orator speaks. He must understand the possibilities of moods in order to rouse them and guide them aright (Heidegger 1962).

With this in mind, if we understand the words of the delusional person as coming from a particular mode of attunement, then careful attention to their words might allow the interlocutor to arrive at an intimation of the mode of attunement the delusional person inhabits. Any attempt to help the person will require re-establishing acceptance and this can only be done through an attempt to reconstruct how their present mood shapes their picture of reality. Without the proper acknowledgement of their situation, the person with the delusion will feel that he or she is not being understood and so is likely to withdraw from conversation.

In terms of overcoming delusions, one particular study by Giannini (2001) seems relevant. Giannini talks about his use of fiction in getting adolescents to open up about fantasy worlds in which they may be thinking through real world social problems. The young adults he saw were not psychotic as they could distinguish these worlds from the real world. The therapy involved getting the young adults to read a book as a focal point for discussion that centred around the book’s plot and characters:

Gradually, connections and identifications developed between these patients and the fictional characters. The ever-mutable fantasies and fixtures of the patient’s inner world were inexorably translocated to World of Tiers [the book series which Giannini instructed the patients to read]. Stable fantasies generated stable symbolisations, and the symbolic language through which we communicated finally became comprehensible. With this common medium, misunderstanding decreased and mutual trust grew (Giannini 2001).

The relevance of this study to the situation of a person with delusions is that fiction and poetry provide a point of focus for a discussion where reality is bracketed and so enables, as Giannini says, a neutral context in which mutual acceptance can be established. Particularly pertinent to what has been argued in this paper is that entering a fictional world through reading would possibly allow the delusional person to escape the all-encompassing nature of their own delusional mood and give them an alternative to their own picture of reality. Of course fiction is an excellent way of altering someone’s mood. It would be useful if the person could relate their own situation to the characters in a book as a discussion of this could give an interlocutor a better understanding of the delusion. However, it is possible that the fiction itself may become part of the delusion, in that it might be taken to contain hidden messages and, of course, especially in schizophrenia, people can have problems with focussing their attention. The problem with attention could be dealt with by watching a film instead, which could equally provide a context and a focus for discussion. Or another suggestion could be that sufferers from delusions could be encouraged to read poetry as a way of understanding how words can depict a mood but also come to an understanding that the mood itself can be reflected upon in a more tranquil mood defined by acceptance. The incorporation of the fictional content of a novel, a poem or a film into a person’s delusion could itself be discussed and used as a key for understanding the person and the way the mood of the delusion changes in relation to what is going on in the person’s environment. The key point to be taken from this study is how a fictional context for using words can give the sufferer from delusions the space to reflect on their own mooded articulation of the world and see how it is possible to move between moods in our comprehension of the world. If this is done in the safe space of therapy it might open up the possibility for the person suffering from delusions to use this insight in everyday life.

Finally, the last point to be made in this section, is based on Wittgenstein’s claim that his form of ‘therapy’ tries to, ‘bring words back from their metaphysical to their everyday use’ (Wittgenstein 1963). The method used by ordinary language philosophers involves overcoming scepticism by focussing on our actual use of language in everyday contexts. The way this could be applied to delusions might be to try to get the delusional person to write poetry about their delusion. In such a way it might be possible to re-establish an ‘as if’ stance to their delusions by allowing them to give their mood its full reign in an appropriate context. Again, the product of such writing may help others to understand the person, which would be an end in itself. Also, objectifying their delusion in a poem may enable the person suffering the delusion to obtain a distance from the delusion by having it independent of them in writing. Again the key insight for the person to gain is that they need not be trapped by a mood e.g. of paranoia, but can express a mood and then reflect on it from a mood of acceptance. Support for this claim is given in a study by Bernard et al. (2006) where people who had suffered from psychosis were asked to write about the most stressful aspects of their experience and treatment when psychotic. It was found that this helped reduce symptoms of trauma surrounding the psychosis in comparison with others who did not write about it. This would suggest that writing about the delusional experience (and especially given the freedom of form in writing a poem) could help the person to come to terms with the experience and come to an external viewpoint on their delusions opening up space for them to let go of those delusions. Pennebaker (1997) provides evidence for both the general psychological (as well as health and intellectual) benefits that can accrue from the written disclosure of emotionally traumatic events. The reason for such effects is not well understood at present, but my arguments about the necessity of expressing a mooded apprehension of the world to return to a mood of acceptance might go some way towards providing a rationale.

## Conclusion

In this paper I have put forward the claim that poetry might be one key for understanding the articulations of those suffering from delusions. I argue that poetry is one way of ‘wording the world’ from a particular mood, but that it is open to the poet to reflect on the mood they write about from the mood of acceptance and so see their language as non-literal. For the sufferer from delusions this move seems to be unavailable as they are not able to access a mood of acceptance. I backed up these claims by looking at the moods that philosophers, exemplified by Hume, write in and their strategies for dispelling those moods and so remaining in the everyday. I looked at one competing theory of delusions and after analysis found that my view can provide a more comprehensive understanding of delusions. Finally taking the claims of this paper further I looked at directions for using poetry in helping sufferers overcome delusions. The view enumerated here suggests that more work should be done on looking at similarities between how poets ‘word the world’ and how delusions are understood by sufferers and that mood and basic emotions hold the key to developing an understanding in this area. A final note would be that conditions that include delusions as a symptom could have the stigma surrounding them dispelled (however ridiculous this may sound to the adherent of the biomedical approach) by viewing delusions as a form of poetry.

## Footnotes

1 This quote is taken from a book by Torrey (2001)

2 I take the mood that allows us to contemplate other moods as being one of acceptance as this is the mood Cavell states that we need to overcome to the skeptical mood. ‘Whereas what the sceptic suggests is that since we cannot know the world exists, its presentness to us cannot be a function of knowing. The world is to be *accepted*…’ (Cavell 1976)

3 ‘A mood assails us. It comes neither from “outside” nor from “inside” but arises out of Being-in-the-world, as a way of such Being’ (Heidegger 1962).

4 A more detailed argument for this position is made by Cavell (1981) and Mulhall (1997). They call this idea an ‘epistemology of moods’.

5 By ‘atmosphere’ here I am indicating that the mood permeates the relation between the person and their environment as a whole rather than a specific object in the environment.

6 I am using the term ‘ground mood’ in the sense that Heidegger in *What is metaphysics?* (Heidegger 1993) calls anxiety a ‘grundstimmung’. That is, as a fundamental mood that provides an absolutely basic framework of meaningfulness through which a world can show up. Acceptance is a basic mood in the sense that it underlies our ability to orientate according to commonsense. Here I claim that someone experiencing psychosis is unable to return to this basic mood.

7 My position would not be undermined if such damage to a sub-personal capacity were found and would in fact suggest the phenomenology of such damage for the person suffering from the delusion.
